# K_ATP _channel subunits in rat dorsal root ganglia: alterations by painful axotomy

**DOI:** 10.1186/1744-8069-6-6

**Published:** 2010-01-26

**Authors:** Vasiliki Zoga, Takashi Kawano, Mei-Ying Liang, Martin Bienengraeber, Dorothee Weihrauch, Bruce McCallum, Geza Gemes, Quinn Hogan, Constantine Sarantopoulos

**Affiliations:** 1 Department of Anesthesiology, Medical College of Wisconsin, 8701 Watertown Plank Road, Milwaukee, WI 53226, USA; 2 Universitätsklinik für Anästhesiologie und Intensivmedizin, Universitätsklinikum Graz, Austria; 3 Department of Pharmacology & Toxicology, Medical College of Wisconsin, 8701 Watertown Plank Road, Milwaukee, WI 53226, USA

## Abstract

**Background:**

ATP-sensitive potassium (K_ATP_) channels in neurons mediate neuroprotection, they regulate membrane excitability, and they control neurotransmitter release. Because loss of DRG neuronal K_ATP _currents is involved in the pathophysiology of pain after peripheral nerve injury, we characterized the distribution of the K_ATP _channel subunits in rat DRG, and determined their alterations by painful axotomy using RT-PCR, immunohistochemistry and electron microscopy.

**Results:**

PCR demonstrated Kir6.1, Kir6.2, SUR1 and SUR2 transcripts in control DRG neurons. Protein expression for all but Kir6.1 was confirmed by Western blots and immunohistochemistry. Immunostaining of these subunits was identified by fluorescent and confocal microscopy in plasmalemmal and nuclear membranes, in the cytosol, along the peripheral fibers, and in satellite glial cells. Kir6.2 co-localized with SUR1 subunits. Kir6.2, SUR1, and SUR2 subunits were identified in neuronal subpopulations, categorized by positive or negative NF200 or CGRP staining. K_ATP _current recorded in excised patches was blocked by glybenclamide, but preincubation with antibody against SUR1 abolished this blocking effect of glybenclamide, confirming that the antibody targets the SUR1 protein in the neuronal plasmalemmal membrane.

In the myelinated nerve fibers we observed anti-SUR1 immunostaining in regularly spaced funneled-shaped structures. These structures were identified by electron microscopy as Schmidt-Lanterman incisures (SLI) formed by the Schwann cells. Immunostaining against SUR1 and Kir6.2 colocalized with anti-Caspr at paranodal sites.

DRG excised from rats made hyperalgesic by spinal nerve ligation exhibited similar staining against Kir6.2, SUR1 or SUR2 as DRG from controls, but showed decreased prevalence of SUR1 immunofluorescent NF200 positive neurons. In DRG and dorsal roots proximal to axotomy SLI were smaller and showed decreased SUR1 immunofluorescence.

**Conclusions:**

We identified Kir6.2/SUR1 and Kir6.2/SUR2 K_ATP _channels in rat DRG neuronal somata, peripheral nerve fibers, and glial satellite and Schwann cells, in both normal state and after painful nerve injury. This is the first report of K_ATP _channels in paranodal sites adjacent to nodes of Ranvier and in the SLI of the Schwann cells. After painful axotomy K_ATP _channels are downregulated in large, myelinated somata and also in SLI, which are also of smaller size compared to controls.

Because K_ATP _channels may have diverse functional roles in neurons and glia, further studies are needed to explore the potential of K_ATP _channels as targets of therapies against neuropathic pain and neurodegeneration.

## Background

ATP- sensitive potassium (K_ATP_) channels are octamers made of four pore-forming inward rectifiers (Kir) that co-assemble with four regulatory sulfonylurea receptor (SUR) subunits [1,2]. Different tissue-specific subunit combinations have been identified that include Kir6.2/SUR1 (pancreatic), Kir6.2/SUR2A (cardiac), and Kir6.1(or Kir6.2)/SUR2B (vascular smooth muscle) subtype.

K_ATP _channels are inhibited by physiological [ATP]i, and are activated when the intracellular ADP/ATP ratio increases as a result of energy depletion secondary to hypoxia, ischemia or metabolic stress [3,4]. Their activation results in K^+ ^efflux, leading to membrane hyperpolarization, decreased excitability, attenuation of transmitter release, and protection from cell death in heart [5] and brain [2]. K_ATP _channels also act as transducers and effectors of neuronal preconditioning [6].

We have recently reported that DRG neurons express K_ATP _currents that decrease after painful nerve injury [7-10]. The loss of K_ATP _current contributes to neuropathic pain through increased membrane excitability, amplified neurotransmitter release [9], and possibly increased susceptibility to cell death. Despite the importance of K_ATP _channels in normal and pathological function, their expression has not been investigated in peripheral sensory pathways. Therefore, we characterized the distribution of the subunits of K_ATP _channels in DRG, and identified alterations in their expression and distribution following painful nerve injury.

## Methods

### Animals

All procedures were approved by the Animal Care and Use Committee (IACUC) of the Medical College of Wisconsin.

### Induction of Experimental Neuropathic Pain

We randomized male Sprague-Dawley rats (120-140 g) by chance either to a spinal nerve ligation (SNL) or to a control group. All surgical procedures were performed under general anesthesia with isoflurane 1.5-2% in O_2_. Ligation of the lumbar 5^th ^and 6^th ^spinal nerve was performed by the technique of Kim and Chung [11], as we have described previously [8-10].

### Behavioral Testing, Tissue Harvesting and Cell Dissociation

Neuropathic pain following SNL was confirmed by behavioral testing for ipsilateral mechanical hyperalgesia at the 10^th^, 12^th ^and the 14^th ^day after surgery [8,12]. After 30 min of rest we applied the tip of a 22 gauge spinal needle on the plantar surface of both hind paws in random order with pressure adequate to indent but not penetrate the plantar skin. Each needle application produced either a normal brief reflexive paw withdrawal, or a hyperalgesia-type response that included sustained (> 1 s) paw lifting, shaking, and grooming [12]. Rats with probability of hyperalgesia ≥ 20% were considered as expressing neuropathic pain phenotype. These constituted 81% of the animals subjected to SNL. We determined that this threshold of 20% ensures optimal sensitivity and specificity for detecting neuropathic pain phenotype after SNL based on analysis of receiver operating plots from previous studies [12].

Dorsal root ganglia were harvested between the 17^th ^and 28^th ^day after control or SNL operation, from rats euthanatized by decapitation under deep isoflurane anesthesia. For the electrophysiological experiments and confocal microscopy studies, we dissociated DRG neurons as described previously [8].

### RT-PCR for K_ATP _Channel Subunits

Total mRNA was isolated from neurons dissociated from one L5 control DRG at each time. For cell isolation each ganglion was enzymatically dissociated in 0.5 ml DMEM/F12 (Dulbecco's modified Eagle's medium F12; Gibco, Invitrogen Corp., Carlsbad, CA) with 0.025% w:v liberase blendzyme 2 (Roche Diagnostics Corp., Indianapolis, IN) for 30 min in an incubator at 37°C. After centrifugation and removal of the supernatant, a second incubation at 37°C followed for another 30 min in 0.25 ml DMEM with 0.0625% trypsin from bovine pancreas (10,000-15,000 BAEE units/mg protein; Sigma, St. Louis, MO; Cat.No. T8802) and 0.0125% deoxyribonuclease 1 from bovine pancreas (≥ 2,000 Kunitz units/mg protein; Sigma, St. Louis, MO; Cat.No. D5025) in 0.25 ml DMEM.

After adding 0.25 ml trypsin inhibitor 0.1% w:v (Sigma), cells were centrifuged (600 rpm for 5 min). For RNA isolation cells were treated with TRIzol reagent (Invitrogen Life Technology) following the supplier's protocol. Isolated RNA was incubated with DNAse (Ambion, Austin, TX) to eliminate DNA contamination, and then 1.2 μg RNA was used for cDNA generation using the Retroscript Synthesis Kit (Ambion). The segment of interest was reverse-transcribed for 2 hours at 42°C.

PCR was performed with 1.5 μl cDNA in total 25 μl using specific primers for Kir6.1 [forward (fw): GGATAATCCCATCGAGAGCA, reverse (rv): CTCAGCCACTGACCTTGTCA), Kir6.2 (fw: TCCAACAGCCCGCTCTAC, rv: CAGCGTTTTGTCCCCATC), SUR1 (fw: TGAAGCAACTGCCTCCATC, rv: GACAAGCCGGAAAAGCTTC) or SUR2 (fw: ACCTGCTCCAGCACAAGAAT, rv: CCTGGTCATTGTGATGAAGAGA)] and Taq DNA polymerase (Roche). The 35 cycles included initial denaturation at 94°C, 4 min; 94°C, 10 sec; 56°C, 30 sec; 72°C, 2 min; and final extension 72°C, 10 min. In negative control experiments reverse transcriptase was omitted. PCR products (12 μl) were separated by electrophoresis on a 1.5% agarose gel stained with ethidium bromide and visualized with a Molecular Imager (Bio-Rad, Hercules, CA). The analysis was repeated with neurons dissociated from DRG of three different control rats.

### Western blotting

Dorsal root ganglia, brain and pancreas from control rats were immediately frozen in liquid nitrogen after removal and stored in -80°C. After thawing in ice, they were homogenized in 100 μl lysis buffer (in mM: 20 MOPS, 2 EGTA, 5 EDTA, 30 NaF, 40 β-glycerophosphate, 10 Na-pyrophoshate, 0.5% NP-40). Samples were prepared for SDS-PAGE by 1:1 dilution with Laemmli buffer, and 30 μg of protein was loaded in each well. Staining with Ponceau S solution was used to verify protein transfer and protein loading to a PVDF membrane. Blots were incubated with 5% non-fatty dry milk (NFDM), 3% bovine serum albumin in 0.1% TBS-T solution for 1 h and then with antibodies (1:200) against SUR1, SUR2, Kir6.2 and Kir6.1, in 1% NFDM overnight at 4°C. Three 15 min washes in TBS-T followed before staining with each secondary antibody (goat anti rabbit HRP-IgG, Santa Cruz 1:10.000) for 2 h, at room temperature. The ECL-Plus chemiluminescence detection kit was used to detect the antigen-HRP conjugated antibody complex (GE Healthcare, Amersham, PA). Each determination was performed 3 times with tissue from different animals.

### Immunohistochemistry

Dorsal root ganglia were cryoprotected in 4% PFA with 15% sucrose in 0.1 M PBS for 1 h, followed by 30% sucrose in 0.1 M PBS overnight. Subsequently DRG embedded in Tissue-Tek^® ^OCT, were sectioned (5 or 12 μm) with a Leica cryostat (Jung CM 1800; Vienna, Austria), plated onto Gatenby's solution subbed slides, and post-fixed in 4% PFA with 4% sucrose. After blocking with 4% normal goat serum for 1 h at room temperature, slides were incubated in polyclonal rabbit antibodies to Kir6.1, Kir6.2 (1:500, Alomone, Jerusalem, Israel), and SUR1 or SUR2 (1:50, Santa Cruz Biotechnology, Santa Cruz, CA). Some samples were also co-labeled with antibodies against neurofilament 200 (NF 200; 1:1000, Abcam, Cambridge, UK) and calcitonin-gene related peptide (CGRP; 1:50; Santa Cruz). The nodes of Ranvier were identified with contactin-associated protein 1 (Caspr, 1:500; clone K65/35, UC Davis/NINDS/NIMH NeuroMab Facility, University of California, Davis, CA 95616), which is localized in paranodal sites [13,14]. Samples were incubated at 4°C overnight. After series of washes, samples were stained with goat anti-rabbit Texas Red (1:500, Jackson Immunoresearch, USA), or goat anti-rabbit Alexa 546 (1:500, Molecular Probes, USA), or goat anti-mouse Alexa Fluor 488 (1:1000, Molecular Probes, USA) as secondary antibodies. DAPI (1:1000; Sigma, St Louis, MO, USA) was used for nuclear staining to distinguish neuronal somata from satellite glial cells. All antibodies were diluted in 1 × GDB containing 0.3% Triton. Slides were mounted with Shur/Mount medium and cover-slipped. For negative controls, only the secondary antibodies were applied. Rat pancreas, brain, and vascular tissues were used as positive controls.

To identify that the pore forming Kir6.2 subunits co-localize with the regulatory SUR1 subunits in DRG as they do in other tissues, we co-labeled some samples with antibody against Kir6.2, with FITC conjugated-glybenclamide (ER-Tracker Green BODIPY-Glybenclamide, Molecular Probes) [15], and with DAPI. Specifically, frozen sections treated with anti-Kir6.2 were incubated with BODIPY-Glybenclamide (20 nM) in 0.1 M PBS for 60 min in room temperature, followed by two washes and DAPI staining. For negative controls, slides were incubated with 1 μM unlabeled glybenclamide for 30 min in room temperature prior to addition of 20 nM FITC conjugated-BODIPY-glybenclamide for 60 min.

Images were obtained using a digital camera and the Spot Diagnostics Instruments software (Version 4.5 for Mac OS X) through a Nikon Eclipse E600 (Nikon, Japan) microscope. Confocal microscopy employed a Nikon Eclipse TE 200-U (Nikon, Japan) equipped with EZ C1 laser scanning software. Images were captured using a 30 μm emission pinhole with a 40× objective.

The criteria used in analysis were consistent throughout the study of all images. The inspection and analysis of samples was performed by the same investigator, who was blinded to the groups of the animals the samples were obtained from. The prevalence and intensity of immunofluorescence in somata and nerve fibers were measured using the Metamorph (Version 7.1; Downingtown, PA). The intensity of fluorescence within cell targets was compared to background, as has been previously used in DRG immunostaining analysis [16,17]. Target was considered positive when intensity in area of interest was threefold greater than in background. This was confirmed by subjective evaluations of randomly chosen images. By this way, there was a consistent agreement between areas considered positive by direct visual inspection and areas exhibiting intensity/background ratio >3. Only cells with clearly visible nuclei were selected for further analysis. We measured the areas of the neuronal somata using the ImageJ software (Java, version 1.5.0-13; Wayne Rasband, Research Services Branch, NIH, Bethesda, MD).

### Electron microscopy (EM)

For preparing samples for immuno-EM, freshly dissociated rat DRG tissues were high pressure frozen, freeze substituted in ethanol and 0.5% uranyl acetate, and embedded in Lowicryl K11 M resin. Ultrathin sections were prepared on formvar/carbon coated grids and incubated with anti-SUR1 or anti-Kir6.2 antibodies, washed, and then labeled with goat anti-rabbit antibodies conjugated to 10 nm colloidal gold. Sections were examined in a Hitachi H600 TEM 9 (Hitachi, Japan) and images recorded using an AMT 1K camera (Danvers, MA).

For preparing samples for measuring SLI by EM, a mixture of 2% glutaraldehyde and 4% PFA in 0.1 M sodium cacodylate buffer (pH 7.2) containing 0.5 mM CaCl_2 _was applied onto exposed rat DRG in situ. After approximately 30 s, fixed pieces of the DRG and nerve were excised and placed into fresh fixative (as above) and fixed for a further 1 h on ice. Tissues were then washed in ice-cold buffer, 3 × 5 min changes, then post fixed with 1% osmium tetroxide and 1.5% potassium hexacyanoferrate for 2 h on ice. Following post fixation, the tissues were rinsed 3 × 5 min in distilled water, dehydrated through graded methanol and embedded in Embed 812 resin. Ultrathin (60 nm) sections were cut, stained with saturated uranyl acetate in 25% ethanol followed by Reynolds lead citrate and examined in a JEOL 2100 TEM (Japanese Electron Optics, Ltd). The areas of SLI were measured using the ImageJ software by an investigator blinded to the experimental group. For consistency reasons, we measured only incisures that traversed the whole width of the myelin sheaths (complete incisures) [18]. Only longitudinal tissue sections were obtained for analysis. We examined sections from three separate sampling sites: from the L5 spinal nerve proximal to ligation, from within the DRG, and from the dorsal root proximal to DRG. The investigator who captured the images of SLI was blinded to the experimental group.

### Electrophysiological recordings

We used electrophysiological recordings to further verify the specificity of our antibody against the SUR1 subunit, and to confirm that staining is localized in the plasmalemmal membranes of neuronal somata. We tested whether the anti-SUR1 antibody affected the blocking effect of glybenclamide on K_ATP _single-channel currents recorded using the excised inside-out configuration of the patch-clamp technique. For this reason neurons from control (SS) rats were preincubated with anti-SUR1 antibodies (1:60) for 2 h and compared with neurons preincubated in control solution without antibody at 37°C for the same time. Single-channel K_ATP _currents were recorded from large diameter neuronal somata at room temperature (20-25°C) as described previously [8-10]. Glybenclamide, a selective K_ATP _channel blocker [19], was obtained from Sigma and diluted daily from 50 mM stocks in DMSO kept at 4°C. The bath (intracellular) solution contained (in mM): 140 KCl, 10 Hepes, 5 EGTA, and 2 MgCl_2_. The pipette (extracellular) solution contained (in mM): 140 KCl, 10 Hepes, 10 d-glucose, and 0.5 EGTA. The pH of all solutions was adjusted to 7.4 with KOH. Membrane potential was clamped at -60 mV. A conventional 50% current amplitude threshold level criterion was used to detect open events. Channel open probability (Po) was determined from the ratios of the area under the peaks in the amplitude histograms fitted by a gaussian distribution. Channel activity was calculated as NPo, where N is the number of observed channels in the patch, from data samples of 30 s recordings duration in the steady state. Mean open time values were estimated from patches that contained predominantly one type of single channel opening. The NPo of the K_ATP _channels was normalized to the baseline NPo value obtained before the drug at bathing solution without ATP, indicating relative channel activity (relative NPo).

### Statistical analysis

Data were compared between control and SNL DRG, or between NF200 positive or negative neurons by Student's or Fisher's exact tests, as appropriate, using the SPSS 16.0 for Mac statistical software (SPSS Inc, Chicago IL). Concentration-response curves were plotted using the Prism 4 for Macintosh (GraphPad Software, Inc) and compared using ANOVA. Areas of SLI were also compared between groups and sites of sampling by ANOVA, using the univariate general linear function of the SPSS, followed by Bonferroni post hoc tests or Student's t test whenever appropriate.

## Results

We used 16 control rats with probability of hyperalgesia scores 0 ± 0% and 9 SNL rats with 42 ± 20% respectively (p < 0.001 versus control).

### Expression of K_ATP _subunits at the mRNA level in DRG of control rats (Fig. 1A)

**Figure 1:**
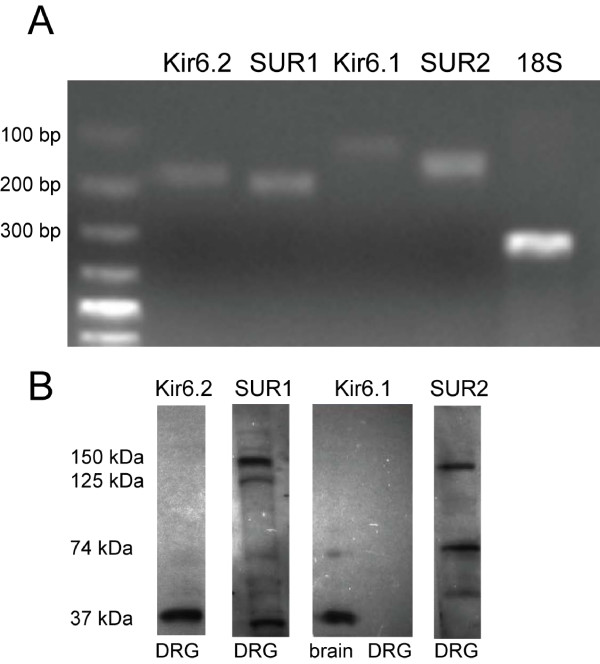
**Expression of K**_**ATP **_**channel subunits in DRG at the mRNA and protein level**. **A**. *RT-PCR product bands consistent with the presence of mRNA encoding K_ATP _channel subunits in DRG from control rats*. Total mRNA was isolated from the L5 DRG. Amplified products appeared at positions corresponding to the expected base pair lengths of 168 (Kir6.2), 182 (SUR1), 110 (Kir6.1), 124 (SUR2) and 315 (18S). **B**. *Western blotting using antibodies against K_ATP _channel subunits in DRG of control rats*. Kir6.2 antibody recognized one immunoreactive band at 37 kDa. SUR1 antibody revealed prominent immunoreactive bands at 150 and 37 kDa, and a less pronounced band at 125 kDa. The band at 150 kDa indicates glycosylated SUR1 bound to Kir6.2. Kir6.1 did not detect any band in DRG tissue, while a band of 37 kDa was detected in samples of brain tissue. Two immunoreactive bands at 150 and 74 kDa were detected by SUR2 antibody. Molecular size markers are shown on the left.

The mRNA for all K_ATP _channel subunits was detected in L5 DRG from control rats. Bands of the expected sizes were detected with primers amplifying Kir6.1 (110 bp), Kir6.2 (168 bp), SUR1 (182 bp) and SUR2 (124 bp) (Fig. 1A). Our negative control experiments, where the reverse transcriptase was omitted, produced only faint nonspecific bands of cDNA, excluding DNA contamination (data shown in Additional File 1: Figure S1).

### Expression of K_ATP _subunits at the protein level in DRG of control rats (Fig. 1B)

In DRG tissue, Kir6.2 antibody recognized one immunoreactive band of 37 kDa, at the expected size of Kir6.2 [20-22]. With antibody against Kir6.1, no bands were detected in DRG tissue, while a band of 37 kDa was found in samples from brain tissue [20,22]. In DRG tissue, the SUR2 antibody detected two expected bands, one at 150 kDa, the expected size for full length SUR, and the other at 74 kDa, in agreement with other reported data for splice variants for the SUR2 subunit [20,22,23]. Finally, the SUR1 antibody detected two prominent bands of 150 kDa and 37 kDa, and one less pronounced band at 125 kDa (Fig. 1B). This is also consistent with SUR1 bands identified by other studies [20,24].

### Rat DRG neurons exhibit SUR1, SUR2, Kir6.2 but not Kir6.1 immunofluorescence (Fig. 2)

**Figure 2:**
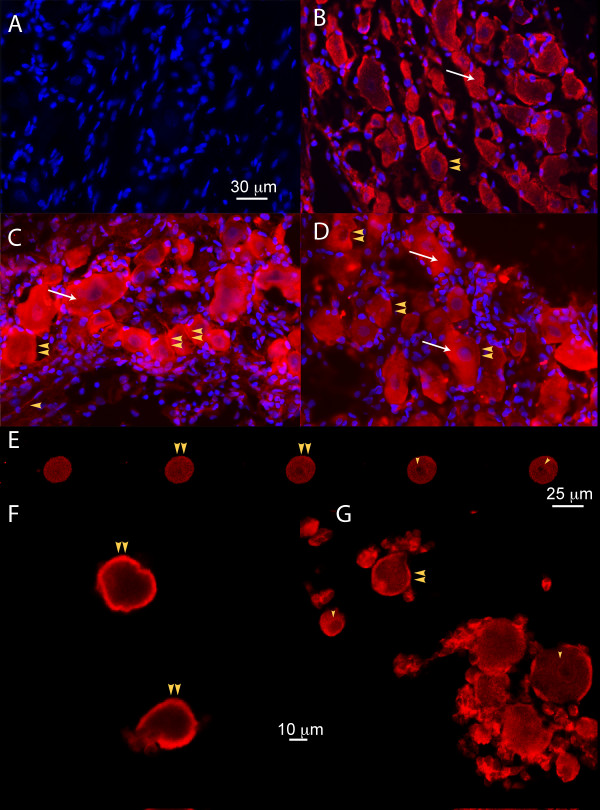
**Presence and distribution of Kir6.2, SUR1, and SUR2 subunits in DRG neurons and satellite glial cells from control rats**. Samples of DRG slices were co-labeled with DAPI, which stains nuclei (blue), and the antibody against each individual subunit (red) (A-D). **A**. Kir6.1 immunofluorescence was absent in DRG. In contrast the same antibody revealed immunostaining in positive controls (rat brain and aorta smooth muscle; not shown). **B**. Immunofluorescence against Kir6.2 is identified on plasma membranes (yellow arrowheads) and cytosol (white arrow). Most satellite glial cells also stained positive for Kir6.2. **C**. Immunofluorescence against SUR1 is observed in the plasma (yellow arrowheads) and nuclear membranes (purple color), as well as along the axons (single yellow arrowhead). Satellite glial cells also stained positive. **D**. Staining against the SUR2 subunit is observed in the plasma membrane (yellow arrowheads), nuclear membrane (purple color), and the cytosol (white arrow). Satellite glial cells also stained positive. In order to confirm the localization of staining in the plasmalemmal membrane of neurons versus the satellite cell membrane, we examined dissociated DRG cells, stained with the same antibodies, using confocal microscopy. These images clearly showed that neuronal plasmalemmal membrane stained positive for SUR1 (**E**), Kir6.2 (**F**), and SUR2 (**G**). Nuclear envelops also stained positive (**E **and **G**, single yellow arrowhead). Distinct positive staining was also observed in satellite cells (**G**). In **E**, images correspond to 5 sequential confocal images of z-projections (with spacing increments of 1 μm).

In negative controls, we observed no immunofluorescence after omitting all primary antibodies. In BODIPY-Glybenclamide negative controls, we also observed no fluorescence after we pre-blocked sections with unlabelled glybenclamide (data not shown). In positive controls, we obtained staining on samples from rat pancreas known to express Kir6.2/SUR1; brain known to express Kir6.2/SUR1 and Kir6.1/SUR1; heart and/or aorta, wherein Kir6.1/SUR2 is expressed [25] (data shown in Additional File 1: Figure S1).

In contrast to positive controls in brain and aorta, no Kir6.1 staining was observed in DRG tissue (Fig. 2A). Kir6.2 immunofluorescence was present in plasma membranes, within the cytosol in a diffuse punctuate pattern, and along the axons (Fig. 2B). In some neurons, staining was also observed in the nuclear envelope.

Most neurons exhibited SUR1 staining in the plasma membrane in a continuous, intense pattern, as well as cytosolic staining that was diffuse and less intense compared to the membrane (Fig. 2C). Anti-SUR1 staining in the cytosol was punctuate, with regional variations of the density of staining. Intense SUR1 staining was also observed on the nuclear envelopes. Finally, SUR2 staining was present in plasmalemmal and nuclear membranes, in the cytosol, and also in axons (Fig. 2D). Most satellite glial cells also stained for Kir6.2, SUR1 and SUR2.

To further distinguish immunostaining localized in the neuronal plasmalemmal membrane from the adjacent satellite glial cells, we studied dissociated DRG cells stained with the same antibodies under confocal microscopy. Neuronal plasmalemmal membrane stained for SUR1 (Fig. 2E), Kir6.2 (Fig. 2F), and SUR2 (Fig. 2G). Neuronal nuclear envelopes (Fig. 2E and 2G) and satellite glial cells also stained positive under these conditions. These images confirmed the presence of separate staining in DRG neurons and in satellite glial cells. Similar findings regarding the distribution of K_ATP _channel subunits immunoreactivity were observed in DRG from rats after SNL.

**Figure 3:**
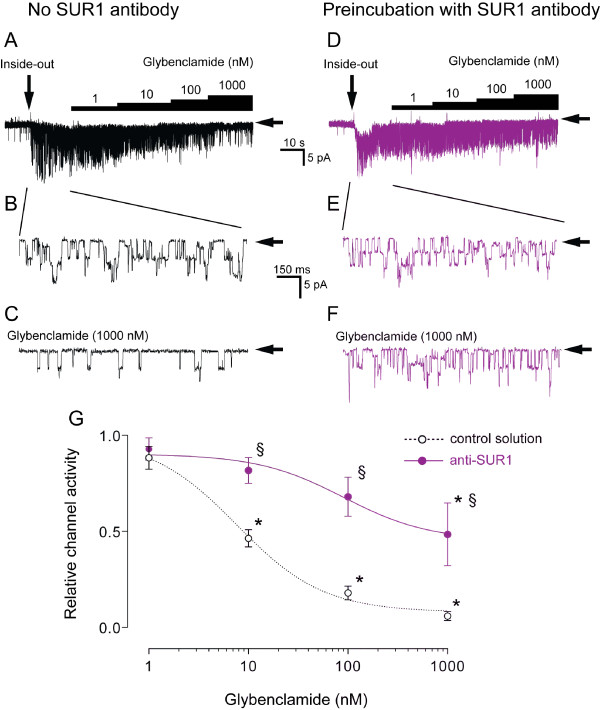
**Preincubation with anti-SUR1 antibody abolishes the blocking effect of glybenclamide on single K_ATP _channel opening in excised membrane patches**. Neurons preincubated with anti-SUR1 antibody (n = 5, **D, E, F**; purple) were compared to neurons preincubated in antibody-free solution (n = 7, **A, B, C**). Horizontal arrows indicate closed channel. **A**. Representative trace of K_ATP _channel activity in patch excised from a neuron preincubated in antibody-free solution. In these neurons glybenclamide inhibited channel activity in a concentration-dependent fashion. **B**. Marked channel activity occurred upon excision of patch (vertical arrow in **A**) into an ATP-free solution. **C**. Glybenclamide 1000 nM blocked channel activity under control conditions. **D**. Representative trace of K_ATP _channel activity in patch excised from a neuron preincubated with anti-SUR1 antibody. **E**. Excision of patch (vertical arrow in **D**) into an ATP-free external solution also activated channel. Cell-free patch exhibited similar K_ATP _single channel activity as in controls (**B**). **F**. In contrast to neurons preincubated in antibody-free solution (**C**), glybenclamide 1000 nM failed to block channel activity after preincubation with anti-SUR1 antibody. **G**. Blocking effect of glybenclamide under control conditions is shown in the concentration-response curve (dotted-line; lower trace). Cumulative application of glybenclamide failed to block channel activity after preincubation with anti-SUR1 antibody, as indicated by the less steep concentration-response curve in **G **(solid line; upper trace). Means ± SD are shown. *:p < 0.05 versus glybenclamide 1 nM; §: p < 0.05 versus control. (Student's t tests were used for intergroup, and Bonferroni tests for intragroup post hoc comparisons).

To test whether the binding target of the anti-SUR1 antibody was part of the actual channel in the neuronal plasmalemmal membrane, and specifically on the regulatory SUR1 subunit, we obtained single channel recordings from excised membrane patches, using the inside-out configuration of the patch-clamp technique, after adding anti-SUR1 antibody in the bath at 37°C for 120 min. As in our previous studies [9,10], marked current activity was observed after patches were excised into a nucleotide-free solution (Fig. 3A, B). In neurons preincubated in control solution without antibody, these currents were inhibited by glybenclamide (1-1000 nM) in a concentration-dependent manner (Fig. 3A, C) within a range which is characteristic for Kir6.2/SUR1 channels. In neurons pre-incubated with anti-SUR1 antibody, excised patch recordings also showed K_ATP _channel activity (Fig. 3D and 3E), but the blocking effect of glybenclamide, which binds to the SUR1 subunit, was significantly suppressed compared to that in the absence of the anti-SUR1 antibody (Fig. 3G). Some small, short lived and infrequent channel openings are also present in the recordings in Fig. 3B and 3E, suggestive of inward rectifiers, but since their activity is limited compared to the dominant K_ATP _current activity, they should not have any overall impact in the analysis. Time-controlled experiments in the absence of glybenclamide resulted in no K_ATP _channel activation or inhibition during the same time periods in both groups (data not shown). These results confirmed that the anti-SUR1 antibody has efficacy against the SUR1 subunit in neuronal plasmalemmal membrane channel.

### Co-localization of Kir6.2 with SUR1 subunits in DRG from control rats (Fig. 4)

**Figure 4:**
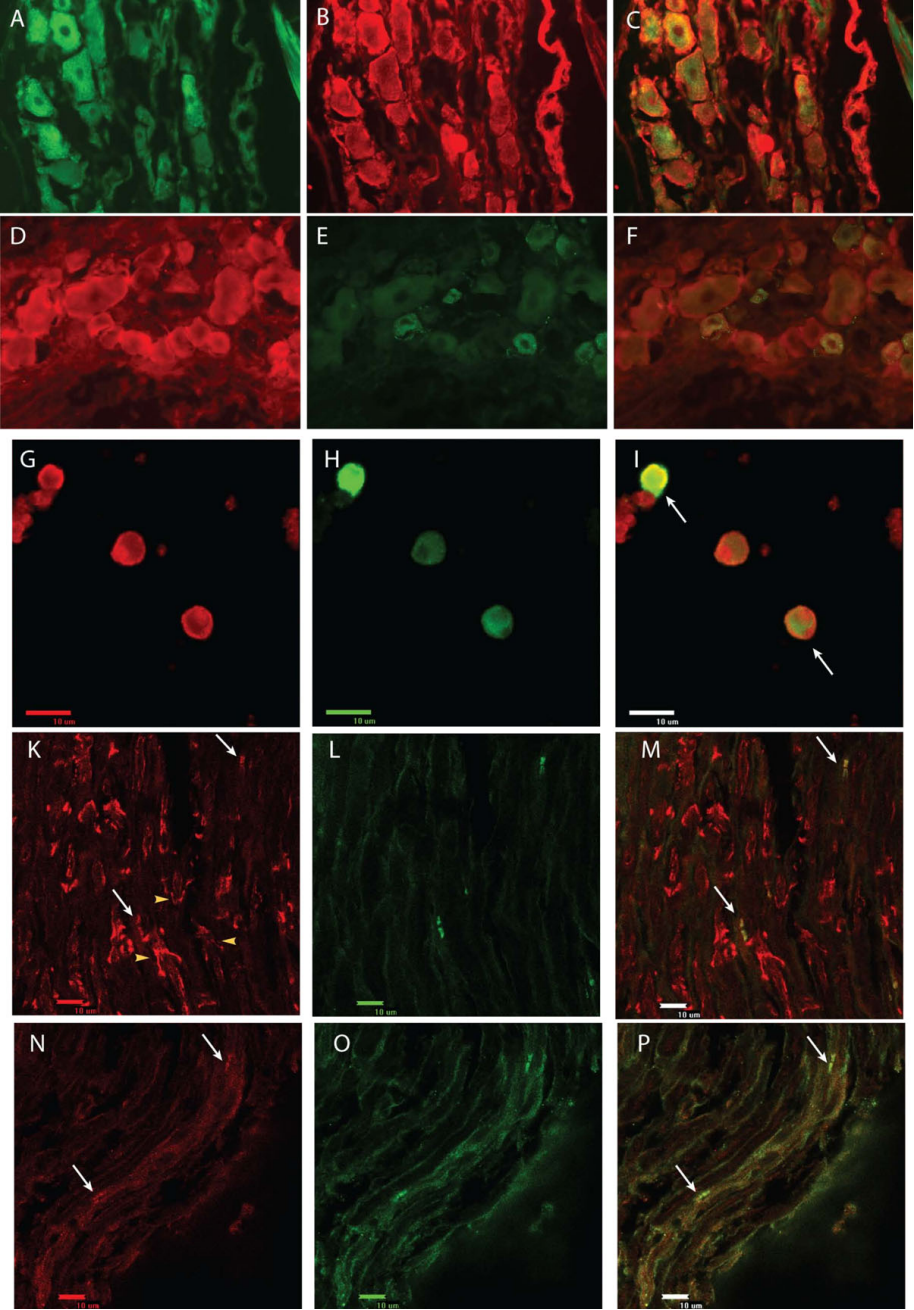
**Colocalization studies in DRG neurons**. A-C. Colocalization of BODIPY-Glybenclamide staining of SUR1 subunits (**A**), with anti-Kir6.2 antibody (**B**), showing that SUR1 subunits are co-expressed with Kir6.2 subunits in the same complexes (**C**: merged). **D-F**. Co-localization of anti-SUR1 antibody (**D**) with anti-CGRP antibody (**E**) in DRG studied under fluorescent microscopy. Merged image (**F**) shows anti-SUR1 immunofluorescence present in small, CGRP positive neurons, as well as in CGRP negative neurons. **G**-**I**. Co-localization of anti-SUR1 antibody (**G**) with anti-CGRP antibody (**H**) in dissociated neurons studied under confocal microscopy. Merged image (**I**) shows SUR1 immunofluorescence present in small, CGRP + neurons (arrows), as well as in CGRP negative neurons. **K**-**M**. Co-localization of anti-SUR1 antibody (**K**) with anti-Caspr antibody (**L**) in DRG studied under confocal microscopy. Merged image (**M**) shows that SUR1 immunofluorescence co-localizes with anti-Caspr staining in paranodal sites. Yellow arrowheads point to SUR1 positive SLI (in **K**). **N**-**P**. Colocalization of anti-Kir6.2 antibody (**N**) with anti-Caspr antibody (**O**) in DRG studied under confocal microscopy. Merged image (**P**) shows that anti-SUR1 immunofluorescence colocalizes with anti-Caspr staining in paranodal sites, adjacent to Ranvier's nodes (White arrows point at paranodal K_ATP _channels). This colocalization of Caspr with SUR1 or Kir6.2 indicates that K_ATP _channels of the Kir6.2/SUR1 subtype are present in paranodal sites (white arrows) adjacent to nodes of Ranvier. All samples are from slices within DRG tissue.

In order to determine whether Kir6.2 and SUR1 subunits co-localize in the same neuronal or glial sites, we further examined the distribution of these subunits in DRG by co-labeling sections with BODIPY-Glybenclamide and anti-Kir6.2. We observed that BODIPY-Glybenclamide staining co-localized with anti-Kir6.2 immunofluorescence in the same sites of the plasmalemmal membrane in DRG neurons (Fig. 4A-C). Combined labeling with BODIPY-Glybenclamide and anti Kir6.2 demonstrated punctuate staining of SUR1 colocalized with Kir6.2 in the cytosol. Additionally, some cells exhibited intense peri-nuclear, ring-like staining (Fig. 4A-C). These findings indicate that Kir6.2 and SUR1 subunits co-localize, apparently forming heteromultimeric K_ATP _channels.

### Localization of SUR1 staining in CGRP positive or negative primary afferent neuronal somata (Fig. 4) from control rats

Anti-SUR1 antibodies stained DRG neurons of different diameters. In order to examine if the population of small nociceptive fibers express K_ATP _channels, we co-labeled ganglia from 3 control rats with anti-SUR1 and anti-CGRP antibodies which specifically stain peptidergic nociceptors [26,27]. The areas of CGRP+ and CGRP- neuronal somata were 420.2 ± 290.6 (range 33.7-1389.3) μm^2 ^and 864.9 ± 389.6 (range 299.1-1719.7) μm^2^, respectively (p < 0.01). We observed that antibody against the SUR1 subunit stained most of the CGRP+ and CGRP- neurons (Fig. 4D-F, Fig. 4G-I). This indicates the expression of K_ATP _channels in CGRP+ peptidergic nociceptors.

### Expression of SUR1 and Kir6.2 subunits in peripheral nerve fibers from control rats (Fig. 4 and Fig. 5)

**Figure 5:**
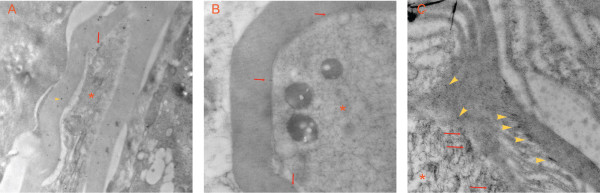
**Distribution of K_ATP _subunits on DRG examined by electron microscopy**. Samples from slices within DRG tissue were treated with antibodies against Kir6.2 or SUR1, which were labeled with gold particles (shown as black dots). **A**. Anti-Kir6.2 staining on axonal membrane (red arrow) and myelin sheath (yellow arrowhead). **B**. Anti-SUR1 staining on axonal membrane (red arrows). **C**. Anti-SUR1 staining on axonal membrane (red arrows) and into a SLI (yellow arrowheads). The axons are marked by red asterisks.

Immunofluorescence against SUR1 and Kir6.2 subunits was also observed along the nerve fibers, in addition to somata. In the case of anti-SUR1, profiles of staining included funnel shaped and rectangular patterns (Fig. 4K). In order to elucidate the distribution of these patterns of staining in reference to nodes of Ranvier, we co-labeled samples with Caspr and SUR1, or Caspr and Kir6.2 antibodies. SUR1 and Kir6.2 were expressed in Caspr positive areas, indicating the presence of K_ATP _channels in paranodal areas (Fig. 4M, Fig. 4P). Additionally, SUR1 fluorescence appeared in funnel shaped profiles in areas apart from Caspr positive sites. This selectively appeared in NF200+ fibers, indicating that K_ATP _channels containing SUR1 subunits are expressed in myelinated sheaths.

**Figure 6:**
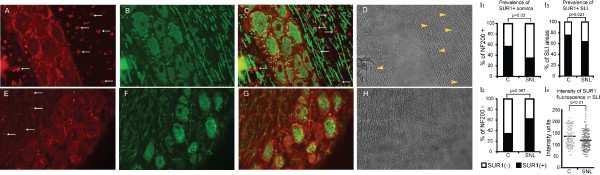
**Distribution of anti-SUR1 immunofluorescence in the subpopulations of NF200+ and NF200- neurons in control (A-D) and SNL (E-H) DRG**. **A, E: **Red anti-SUR1 immunofluorescence is observed on plasma and nuclear membranes, satellite glial cells, and along the peripheral nerve fibers. White arrows point to SLI. These are more intense and funnel shaped in controls (**A**) compared to SNL (**E**), wherein SLI loose their characteristic funnel shape, and appear less intense, thin and disorganized. **B**,**F**. Anti-NF 200 staining (green), distinguishes two DRG neuronal subpopulations, corresponding to larger, myelinated NF200+ and to smaller, non-myelinated NF200- fibers. **C**,**G**. Merged images showing the difference in distribution of SUR1 staining in each neuronal subgroup between SS and SNL DRG. SLI are shown with arrows in **C**. **D**, **H**: Bright field images. SLI are shown by yellow arrowheads in **D**. **I**(**1-4**). Bargraphs showing the differences in the prevalence of SUR1+ staining between control (C) and SNL NF200+ (**I1**) and between control (C) and SNL NF200- neuronal somata (**I2**), compared by Fisher's exact tests. **I3**. Bargraphs showing the decreased prevalence of SUR1+ SLI between control (C) and SNL axons, compared by Fisher's exact test. **I4**. Scatterplots showing the difference in the intensity of SUR1 immunofluorescence in SLI between axons from SNL versus control DRG. Arbitrary fluorescence units are used. Comparisons were made by Student's t test.

To further investigate the distribution of these SUR1 positive funnel-shaped patterns at a higher resolution, we employed EM (Fig. 5) that showed binding of gold-labeled anti-SUR1 and anti-Kir6.2 antibodies on axonal membrane and in the myelin sheath, within sites that are characteristic for Schmidt-Lanterman incisures (SLI) formed by the Schwann cells (Fig. 5C, Fig. 6A). These findings, together with the characteristic microscopic funnel-shaped patterns [18,28], indicate the presence of SUR1-containing K_ATP _channels within SLI.

### Distribution of SUR1 expression in NF200+ and NF200- neurons from control rats, and alterations after axotomy (Fig. 6)

Inspection of our samples suggested that large neurons were more likely to express K_ATP _subunits. This might indicate a cell type-specific expression of K_ATP _channels predominantly in large, myelinated neurons, in agreement with previous electrophysiological data [10]. In order to examine this, we co-labeled DRG sections with antibodies against both SUR1 and NF200, which distinguishes DRG neurons that correspond to large, myelinated subpopulation [26,29]. NF200- neurons correspond to small, unmyelinated C fibers [29,30]. The areas of NF200+ and NF200- neuronal somata from control rats were 1056.5 ± 431.8 (range 418.5-2525.2) μm^2 ^and 385.5 ± 106.9 (range 209.9-677.1) μm^2^, respectively (p < 0.01). In control neurons, SUR1 is expressed at higher rates in NF 200+ somata (34/60, 56.7%) than NF200- somata (10/29, 34.5%; p = 0.07; Fig. 6).

The areas of NF200+ and NF200- neuronal somata from SNL rats were 891.1 ± 250.3 (range 331.3-1392.7) μm^2 ^and 338.12 ± 68.8 (range 229.7-446.4) μm^2^, respectively (p < 0.01). In axotomized neurons from SNL rats, localization and distribution of Kir6.2, SUR1 and SUR2 immunofluorescence was comparable to controls (data not shown). However, only 34.8% of NF200+ SNL somata stained positive for SUR1 compared to 56.7% NF200+ controls (p = 0.02; Fig. 6I1). Within the subgroup of NF200- neurons, the percentage of somata that stained positive for SUR1 (62.5%) was unchanged from controls (p = 0.067; Fig. 6I2).

As shown in Fig. 6I3, fewer SNL axons had SUR1+ SLI (63.8%) compared to control (75.8%; p = 0.021 vs. control). Exposure parameters under which images were captured did not differ between control and SNL axons, allowing us to compare the intensity of fluorescence as well. Intensity of anti-SUR1 fluorescence in SLI was 12% less in SNL compared to control (p < 0.01; Fig. 6I4).

**Figure 7:**
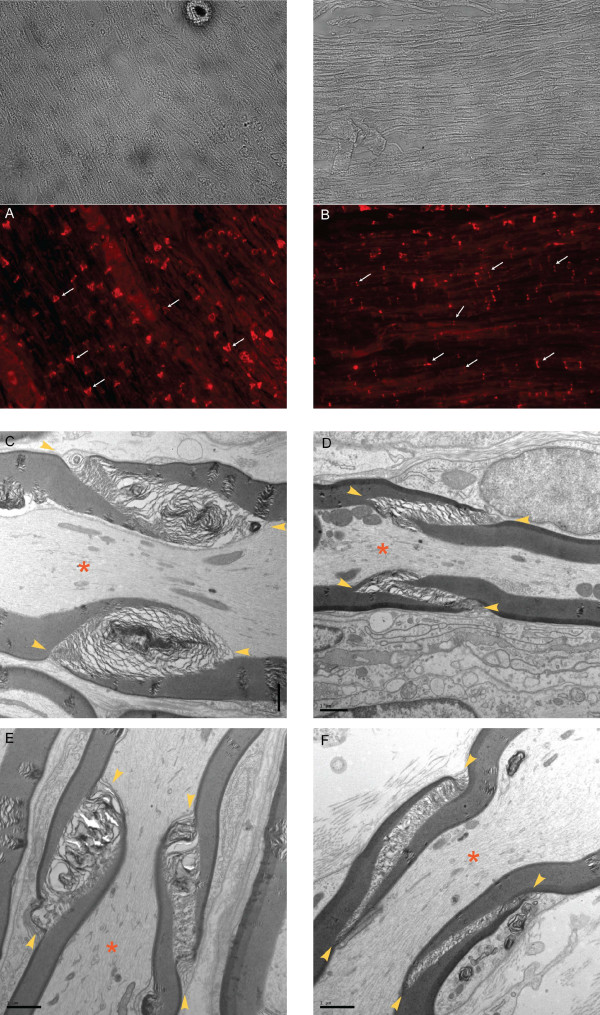
**Differences in SLI between control (A,C,E) and SNL (B,D,F) axons examined by fluorescent and electron microscopy**. **A**. SLI in control DRG. The characteristic funnel-shaped appearance is shown by white arrows. **B**. Following SNL, SLI proximal to axotomy appear disorganized, without the characteristic immunofluorescent funnel shape (as in **A**). Intensity of fluorescence in SLI is lower compared to controls. **C**, **E**. Control DRG samples examined by EM. SLI are shown between yellow arrowheads. **D**, **F**. The areas of SLI (also shown between yellow arrowheads) are decreased proximal to axotomy following SNL, as also presented in details in Table 1. Axons are marked by red asterisks.

The patterns of fluorescence in the SNL axons were also qualitatively different than in control. In SNL, the shapes of SUR1+ SLI were more linear and thin compared to thicker and funnel-shaped SUR1+ SLI in control (Fig. 6A,E). SNL reduced SLI area in peripheral nerves proximal to axotomy, ganglia, and dorsal roots (Fig. 7D, F; Table 1).

**Table 1:** Areas of Schmidt-Lanterman Incisures in peripheral nerves diminish after painful axotomy.

	Area of Schmidt-Lanterman Incisures (μm^2^)		
	Dorsal root	DRG	Peripheral nerve
Control L5	2.7 ± 0.7 (n = 46)	5.3 ± 3.9^*f *^(n = 116)	2.3 ± 1.0 (n = 111)
SNL L5	1.6 ± 0.9* (n = 142)	2.9 ± 2.3*,^*f *^(n = 117)	0.5 ± 0.3* (n = 116)

Areas of Schmidt-Lanterman Incisures measured in electron microscopy sections obtained from three different sites proximal to axotomy, in comparison with similar sites in control DRG neurons. Sampling sites were the dorsal root proximal to DRG, the DRG, and the peripheral nerve distal to DRG but proximal to axotomy. *: p < 0.01 vs control L5 (by Student's t tests). ^*f*^:p < 0.01 vs dorsal root and peripheral nerve (by Bonferroni post hoc tests).

## Discussion

SUR1 or SUR2 subunits always co-assemble with either Kir6.1 or Kir6.2 subunits into functional K_ATP _channel octamers [2,19]. Using immunostaining and Western blots, we showed that peripheral sensory neurons express SUR1, SUR2 and Kir6.2 protein, but not Kir6.1 protein. We identified this co-localization of Kir6.2 with SUR1 subunits by staining with antibody against Kir6.2, and with BODIPY-Glybenclamide, which specifically binds to SUR1 with high affinity at concentrations <40 nM [15]. Our findings from these experiments highlight the presence of K_ATP _channels in control and axotomized neurons. K_ATP _channels in DRG neurons are of the Kir6.2/SUR1 (pancreatic-neuronal) and Kir6.2/SUR2 (cardiac) subtype.

Since suboptimal specificity may limit the reliability of immunostaining techniques [31], we confirmed the specificity of our antibodies by Western blots. The size of the bands that we detected are consistent with those reported in other studies that have identified K_ATP _channel subunits. Furthermore, we functionally confirmed the specificity of our anti-SUR1 antibody by electrophysiological recording, in which antibody application eliminated glybenclamide-sensitive current in excised membrane patches. Use of isoflurane anesthesia as well as the duration of hypoxia may have affected the recordings of potassium currents, as described previously [32]. However, it is unlikely that these factors may have confounded the results because all rats were exposed to the same conditions of anesthesia and the duration of hypoxia. All these procedures were performed by the same investigator in the same fashion and by the same protocol for each animal. Antibodies directed against channel components such as the SUR1 regulatory subunit may alter active sites of the channel or its interaction with ligands, and thus can be used as specific inhibitors [33-35].

Our RT-PCR technique detected mRNA encoding all K_ATP _channel subunits. However, neither immunostaining nor Western blots detected any Kir6.1 at the protein level. Either immunohistochemistry and Western blots were not sensitive enough to detect Kir6.1 protein in DRG neurons, or Kir6.1 mRNA is not translated into protein in these neurons [36,37]. However, the same antibodies did not fail to detect Kir6.1 in brain tissue, which was used as control, indicating competence of our antibodies, and, therefore the lack of translation of Kir6.1 mRNA to protein in DRG neurons.

The biophysical and pharmacological properties of the K_ATP _current recorded in DRG neurons indicate that the predominant membrane channel subtype is the Kir6.2/SUR1, since glybenclamide selectively blocks this subtype in the concentration range used in our experiments [10,19]. Immunostaining against SUR2 implies the presence of non-functional Kir6.2/SUR2 subunits.

We were able to identify the distribution of functional K_ATP _channels and their alterations by axotomy by staining for SUR1 subunits. Previous reports have validated the use of anti-SUR1 only to study the cellular distribution of K_ATP _channels [38]. Identification of SUR1 subunits in membrane and SLI represents post-translational co-expression with pore-forming subunits, because SUR and Kir subunits always combine in functional K_ATP _channels. In contrast, unbound SUR subunits are unstable [39,40].

In addition to neurons, K_ATP _channels are present in glial satellite and Schwann cells, which are known to express K^+ ^currents [41-43]. Since K_ATP _channels are inward rectifiers, our findings are consistent with reports that Schwann cells express inward rectifying K^+ ^currents [44]. Our findings that satellite and Schwann cells express K_ATP _channels also help clarify a controversial issue about the presence of Kir6.2-containing K_ATP _channels in glial cells [45]. K_ATP _channels in these cells, in cooperation with other Kir channels, may convey glial cell-mediated clearance of extracellular K^+^, often termed "K^+ ^spatial buffering" or "K^+ ^siphoning" [45,46].

A consistent finding of our study is the presence of K_ATP _channel subunits in nuclear envelopes of DRG neurons. These channels may regulate gene expression, as have identified in other tissues. Specifically in pancreatic β-cells, K^+ ^fluxes via nuclear K_ATP _channels release Ca^2+ ^stored in the nuclear envelope leading to CREB phosphorylation and gene expression [47]. It is therefore possible that K_ATP _channels in the nuclei of DRG neurons play similar roles.

Kir6.2/SUR1 subunits have not been previously identified in peripheral nerve fibers, although inward rectifying K^+ ^channels have been detected in paranodal sites [42,44,48]. The molecular identity of these inward rectifiers had not been identified previously, but we now show that both SUR1 and Kir6.2 co-localize with Caspr + paranodal areas in axons. This finding implies that K_ATP _channels are the first inward rectifying channels identified in paranodal sites. These sites and the nodes of Ranvier have high metabolic demands [49]. It is thus possible that K_ATP _channels regulate excitability at paranodal sites, and may safeguard these regions of high metabolic activity from injury during energy depletion [50].

Our novel finding that K_ATP _channels are present in SLI, is supported by fluorescent microscopy, and more specifically by EM data, which together confirm the presence of SUR1 subunits in SLI. Schmidt-Lanterman incisures are accumulations of Schwann cell cytoplasm enclosed within the myelin lamellae all along the internodal length of the axon, and also adjacent to nodes of Ranvier [18,28]. These structures contain abundant gap junctions that mediate intra- and intercellular communications [51]. K_ATP _channels regulate opening of gap junctions in other cell types [52], including astrocytes [53], and they may do so in SLI as well. SLI are involved in metabolic functions of the myelin sheath and axon, including growth, maintenance and axonal support [54]. Because of these functions, SLI have high metabolic activity and energy demands, which are elevated following nerve injury [50]. It is thus possible that K_ATP _channels in SLI mediate protection against energy depletion.

Part of the supportive functions of glial satellite and Schwann cells is the synthesis and transfer of ion channels to neighboring axons [43,55]. Indeed, an exchange of K^+ ^channels between Schwann cells and neuronal axons has been shown [56]. Neurons need to supply 500-2000 nodes with channels that undergo rapid turnover. However, because axonal transport from the soma is not always sufficient, especially to the most distant parts of the fiber, Schwann cells may provide these channels to axons through SLI intercellular communications. Therefore, the SUR1 subunits that we have detected in the SLI may be parts of K_ATP _channels in transit towards the adjacent axons, or may be functional constituents of the SLI themselves.

Neurons axotomized by SNL express K_ATP _channel subunits at the same cellular and histologic locations as controls. However after injury, NF200+ somata are less likely to express SUR1 subunits less frequently than control somata. Like other types of K^+ ^channels [57-59], K_ATP _channels decrease after nerve injury, due to their downregulation or to their redistribution to axons.

A novel observation of our study is that the prevalence of SUR1 positive SLI, as well as the intensity of SUR1 immunofluorescence in the SLI, decrease after nerve injury. Our ultrastructural data from EM show that the geometrical area of the SLI after nerve injury is reduced, which may explain diminished SUR1 immunofluorescence. This further indicates disruption of myelin architecture proximal to the site of axotomy, consistent with other reports indicating altered SLI architecture in various peripheral neuropathies [60], including Charcot-Marie-Tooth [51].

Downregulation or decreased K_ATP _channel activity may open gap junctions between SLI, between adjacent Schwann cells, and also between SLI and regenerating axons, as they do in other tissues [52,53]. These open gap junctions in Schwann cells transport ATP and other metabolic substrates to axons [51]. Our observations of decreased SUR1 at these sites after injury suggests that withdrawal of K_ATP _channel activity may support neuronal regeneration.

However, considering the alterations we observed in injured by SNL neurons, dissociated from rats with hyperalgesia, only comparison with neurons from rats subjected to the same treatment (SNL) but not exhibiting hyperalgesia would indicate whether these changes are pertinent in the pathogenesis of neuropathic pain. Thus, rats not exhibiting neuropathic pain phenotype after SNL would be appropriate to serve as an additional control group, the lack of which constitutes a limitation of our study.

We have previously reported the presence of K_ATP _channels in DRG neuronal somata using electrophysiological recordings at the single-channel and whole-cell current level. We now identify the presence of the specific channel subunits that conduct K_ATP _current not only in DRG somata but also in peripheral sensory axons, glial satellite and Schwann cells, including SLI. K_ATP _channel opening is necessary to control excitability and neurotransmitter release in peripheral sensory neurons, as we have previously shown [9]. Another limitation of this and our previous studies is a lack of description of the distribution and role of K_ATP _channels at the presynaptic afferent terminals in the dorsal horns, although their presence at these sites is highly likely [61]. K_ATP _channels may also contribute to neuroprotective functions in DRG as in other neurons [6].

Our previous work has emphasized the role of K_ATP _currents in DRG neurons, which are the primary determinants of sensory function. However, lack of the functional confirmation of K_ATP _currents in glial cells in contrast to DRG neurons is another limitation. Further exploration of the role of K_ATP _channels in glial cell is an important topic, but the necessary studies should include extensive electrophysiological characterization and additional experiments that will be the subject of separate future investigations.

In glial satellite and Schwann cells, K_ATP _channel opening may also support metabolic functions, including K^+ ^siphoning [45]. However, in the peripheral nerves, recovery from injury may also benefit from loss of K_ATP _channels, which facilitates restoration through gap junction opening and facilitated axonal regeneration.

Because of these diverging roles of K_ATP _channels in different neuronal and non-neuronal sites, opposing effects of K_ATP _channel opening in SLI and myelinated nerve fibers after painful nerve injury are likely. However, this is based on speculation. There is a possibility that the effects on one site may override these on another site, therefore further studies are necessary to elucidate the in vivo effects of peripheral application of K_ATP _channel openers for treating neuropathic pain and neurodegenaration.

## Abbreviations

[ATP]i: cytosolic ATP concentration; DRG: dorsal root ganglion; PBS: phosphate buffered saline; PFA: paraformaldehyde; RT-PCR: reverse transcription-polymerase chain reaction; SDS-PAGE: sodium dodecyl sulfate polyacrylamide gel electrophoresis; PVDF: polyvinylidene fluoride; TBS-T: tris buffered saline -tween20; DAPI: 4',6-diamidino-2-phenylindole; GDB: Gelatin Diphosphate Buffer; FITC: Fluorescein isothiocyanate; BODIPY: boron-dipyrromethene; TEM: transmission electron microscope; DMSO: Dimethyl sulfoxide.

## Competing interests

The authors declare that they have no competing interests.

## Authors' contributions

VZ, MYL, DW and BM carried out the immunohistochemical studies. VZ blindly evaluated the images and performed the measurements using Metamorph and ImageJ. TK carried out the electrophysiological experiments and data analysis. VZ, TK, and GG carried out animal surgery and behavior testing. VZ and MB carried out molecular biology experiments. VZ, MYL and CS conceived the study, and participated in its design and coordination. VZ, CS and QH wrote the manuscript. All authors have read and approved the final manuscript.

## Supplementary Material

Additional file 1 **Supplemental Figure Legend**. A. Negative controls for RT-PCR. On negative control experiments, where the reverse transcriptase was omitted, no amplified products appeared at positions corresponding to the expected base pair lengths of 168 (Kir6.2), 182 (SUR1), 110 (Kir6.1), 124 (SUR2) and 315 (18S). B. Positive controls for anti-Kir6.2 antibody (rat brain), anti-SUR1 antibody (rat pancreas), anti-Kir6.1 antibody (rat brain) and anti-SUR2 antibody (mouse cardiac myocyte). All these areas are known to express K_ATP _channels. Bright field images are shown on the right of each image. Additional positive control experiments revealed SUR2 staining in sections from rat aorta (not shown). Nuclei in myocyte are stained by DAPI. Click here for file
