# Supplementary figures and images for: KATP channel subunits in rat dorsal root ganglia: alterations by painful axotomy
Source: Mol Pain. 2010 Jan 26;6:6. doi: 10.1186/1744-8069-6-6 (PMC2825500; doi:10.1186/1744-8069-6-6)

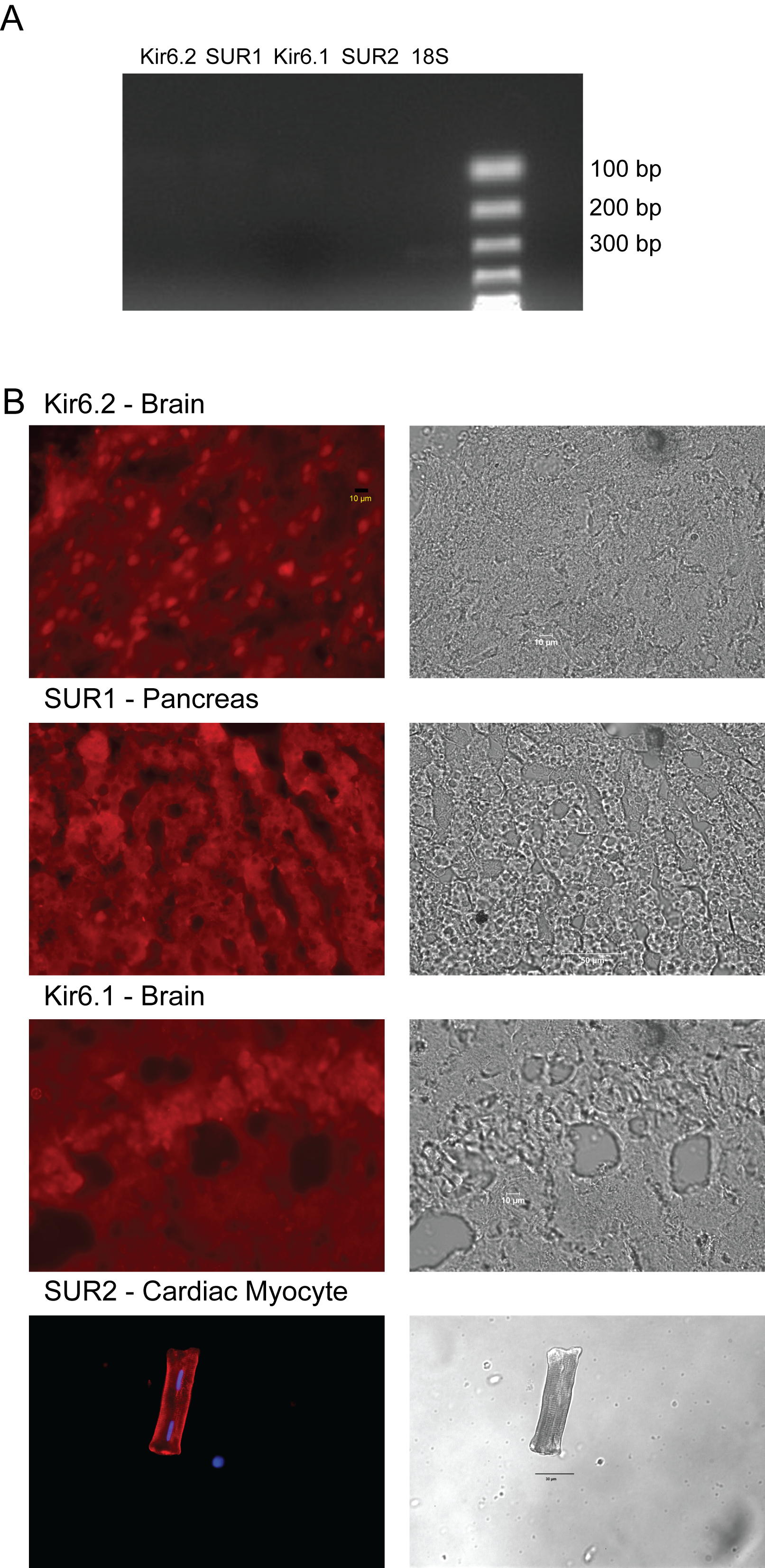

Supplement: Additional file 1 — Supplemental Figure Legend. A. Negative controls for RT-PCR. On negative control experiments, where the reverse transcriptase was omitted, no amplified products appeared at positions corresponding to the expected base pair lengths of 168 (Kir6.2), 182 (SUR1), 110 (Kir6.1), 124 (SUR2) and 315 (18S). B. Positive controls for anti-Kir6.2 antibody (rat brain), anti-SUR1 antibody (rat pancreas), anti-Kir6.1 antibody (rat brain) and anti-SUR2 antibody (mouse cardiac myocyte). All these areas are known to express KATP channels. Bright field images are shown on the right of each image. Additional positive control experiments revealed SUR2 staining in sections from rat aorta (not shown). Nuclei in myocyte are stained by DAPI. [file 1744-8069-6-6-S1.PNG]
